# Conductometric Sensing with Individual InAs Nanowires

**DOI:** 10.3390/s19132994

**Published:** 2019-07-07

**Authors:** Valeria Demontis, Mirko Rocci, Maurizio Donarelli, Rishi Maiti, Valentina Zannier, Fabio Beltram, Lucia Sorba, Stefano Roddaro, Francesco Rossella, Camilla Baratto

**Affiliations:** 1 NEST, Scuola Normale Superiore and Istituto Nanoscienze-CNR, Piazza San Silvestro 12, 56127 Pisa, Italy; 2 Department of Information Engineering, University of Brescia, Via Branze 38, 25123 Brescia, Italy; 3 Department of ECE, George Washington University, Washington, DC 20052, USA (current address); 4 CNR-INO Brescia, Via Branze 45, 25123 Brescia, Italy

**Keywords:** InAs nanowires, gas sensing, electrical transport

## Abstract

In this work, we isolate individual wurtzite InAs nanowires and fabricate electrical contacts at both ends, exploiting the single nanostructures as building blocks to realize two different architectures of conductometric sensors: (a) the nanowire is drop-casted onto—supported by—a SiO_2_/Si substrate, and (b) the nanowire is suspended at approximately 250 nm from the substrate. We test the source-drain current upon changes in the concentration of humidity, ethanol, and NO_2_, using synthetic air as a gas carrier, moving a step forward towards mimicking operational environmental conditions. The supported architecture shows higher response in the mid humidity range (50% relative humidity), with shorter response and recovery times and lower detection limit with respect to the suspended nanowire. These experimental pieces of evidence indicate a minor role of the InAs/SiO_2_ contact area; hence, there is no need for suspended nanostructures to improve the sensing performance. Moreover, the sensing capability of single InAs nanowires for detection of NO_2_ and ethanol in the ambient atmosphere is reported and discussed.

## 1. Introduction

The development of innovative sensors for the detection of chemical and biological species is a crucial ingredient for real advancements in many fields across physical, engineering and life sciences, including healthcare [1,2,3], food safety [4,5], environmental protection [6,7], agriculture [8,9], and security [10]. The use of systems, techniques, and approaches characteristic of nanotechnology to sensing applications is regarded as one of the most promising routes towards more sensitive, selective, fast, stable, and cheap devices [11]. The advent of nanomaterials paved the way to advanced sensor performances, and the continuous progress in nanoscience and nanotechnology holds the promise for large implications in innovative and more performing detection systems [11,12,13,14].

In this context, nanowires (NWs) are emerging as promising platforms for the development of ultrasensitive sensors for the direct detection of biological and chemical species [15,16,17,18]. The large surface-to-volume ratio is considered as one of the key advantages for the increased sensor response of nanowire-based devices [18,19,20,21]. Different NW materials [20,22,23,24], sensed molecules [21,25,26,27], device architectures [28,29,30], and signal transduction methods [31] are currently being studied. The realization of all-electrical devices using semiconducting nanowires as the sensing element is particularly attractive because it enables ready signal transduction and integration into miniaturized systems [15]. The interaction between the NW surface and the adsorbed molecules causes a local modification of the energy band structure in the semiconductor, which affects the electrical transport properties [18]. Nanowires made of different semiconducting materials have been exploited in field effect transistors for sensing applications [20,29,32,33,34], providing useful insights into the understanding of the specific physical mechanisms related to the ability of nanowire devices in detecting different types of analytes.

III-V semiconductor nanowires are well established as a suitable platform for different fields of nanoscience and nanotechnology, including thermoelectrics [35,36], signal transduction [37,38], nanoelectronics, optoelectronics, and plasmonics [39,40,41,42]. Recently, nanowire-based sensing applications have gained enormous interest: InAs NWs, both individual and in array configuration, are considered as promising building blocks for the realization of gas sensors due to their easy growth mechanism, to the high value of the electron mobility and to the likely presence of a surface accumulation layer, which makes them particularly sensitive to the surface state dynamics and the environment [30,43,44,45]. Seminal studies demonstrated their exceptional suitability for humidity and organic vapor detection in an inert atmosphere (either Nitrogen or Helium): while these conditions are far from real applications, key insight on molecule-surface interactions was achieved [46,47,48]. Offermans et al. investigated gas sensing with vertical arrays of nanowires, reporting an impressive response towards concentrations of NO_2_ in N_2_ at room temperature below 100 ppb [30]. They also studied the possibility to functionalize the NW surface in order to improve sensitivity and selectivity [49]. In general, the use of vertical arrays of nanowires is very promising for optical sensing applications [50,51], while for the realization of electrical devices, it displays unease of implementation of electrical contacts. Moreover, NW arrays may suffer from considerable nanowire size dispersion [52,53].

While metal oxide NWs represent one of the most developed classes of nanomaterials for sensing, still one of the main drawbacks remains their relatively high operating temperatures—usually well above 300 K—which may imply a significant power consumption and severely prevent applications in wearable and/or flexible electronics [54]. The sensing mechanism occurring in these nanomaterials can be traced back to the interaction between the gas molecules and the oxygen ionosorbed on the metal oxide surface. It is, therefore, a thermally activated mechanism, and the activation energy typically is larger than 300 K. Thus, usually, metal oxides cannot work at room temperature, unless the activation energy is provided by an external trigger as, for instance, supra-band-gap light excitation [55]. Now let’s consider III-V NWs and specifically InAs NWs. They share with their metal oxide counterpart the characteristic of displaying very high surface-to-volume ratio. Besides, InAs NWs can be grown as single crystals with extremely high control of material properties (e.g., charge carrier type, density, mobility), structure (wurtzite, zincblend, combination of the two, heterostructures), and morphology (length, diameter, tapering) using affordable growth techniques yielding to millions of virtually identical replicas of the same nanostructure, as, for instance, molecular or chemical beam epitaxy methods. On top of this, InAs NWs display an extra advantage: they are characterized by the occurrence of surface charge accumulation layer, ascribable to Fermi level pinning at the surface, which is expected to make these nanostructures very sensitive to the environment. Finally, they are fully compatible with room temperature device operation. In view of all these points, InAs NWs could represent the material platform for a sensing technology alternative to the one based on metal oxide NWs, especially when the room temperature is desirable or mandatory.

The present work aims at investigating the potential of individual InAs nanowires as building blocks for sensor prototypes operating in conditions potentially relevant for real-life applications and provides further insights on the fundamental interaction mechanisms with humidity, NO_2_, H_2_, and ethanol molecules.

## 2. Materials and Methods

The study reports a comparison between two different device geometries, both based on the use of an individual InAs nanowire as the sensing element. In one case, the nanowire is directly drop-casted onto a SiO_2_/Si substrate and supported by it, while in the other one, the nanowire is suspended at approximately 250 nm from the substrate. For simplicity, the first type of device is called “supported”, the latter “suspended”. The interest in this comparison is related to the observation that some types of NW-based sensors display improved performances if a suspended geometry is implemented [28,56]. This improvement was overall attributed to the availability of a bigger portion of the NW surface to gas adsorption and to an enhanced mass transfer and convection around the sensing element. The architectures proposed in the present study have the advantage of very easy transduction, as the detection mechanism is based on the measure of the current response in a two-terminal configuration. Gas sensing tests were carried out towards different gases (humidity, ethanol, H_2_, and NO_2_), by monitoring the source-drain current for both device architectures upon changes in the gas concentration using synthetic dry air as a gas carrier, moving a step forward towards mimicking operational environmental conditions.

Information on the number of sensors that were fabricated, tested, and used for the sensing experiments, as well as on the variance between the samples, can be found in Appendix A.

**Figure 1:**
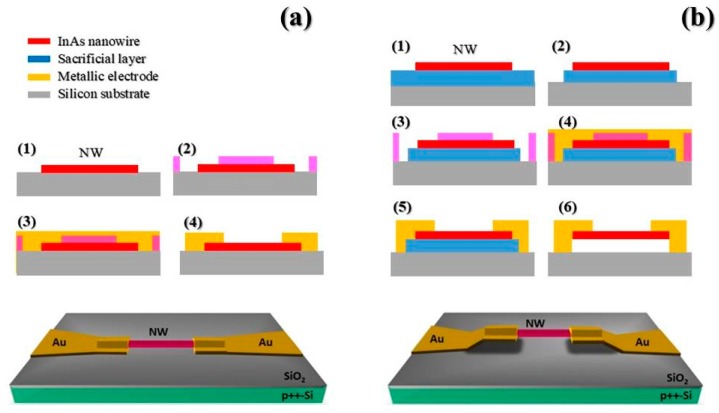
Fabrication protocol for (**a**) supported and (**b**) suspended nanowire-based devices. In the upper part of Figure (**a**) and (**b**), a schematic representation of the fabrication sequence for the two types of devices is depicted. The bottom part shows a schematic representation of the device architectures. The fabrication process for the supported device (**a**) consists of: (1) NW (nanowire) deposition by drop casting, (2) Electron beam lithography (EBL) patterning, (3) metal contact thermal evaporation, (4) Lift-off. The fabrication process of the suspended nanowire device (**b**) includes two additional steps at the beginning of the process, which are the deposition and patterning of a sacrificial layer in the region underneath the nanowire (steps (1) and (2)). The steps (3), (4), (5) coincide with steps (2), (3), and (4) of the case (a). Step (6) consists of the removal of the sacrificial layer by means of O_2_ plasma, leaving the NW suspended at a distance of approximately 250 nm from the substrate.

The basic building blocks of our devices consist of wurtzite n-type InAs nanowires, grown on (111)B InAs substrates, exploiting gold-catalyzed chemical beam epitaxy [52]. The NWs have an average length of 4 µm ± 250 nm and an average diameter of 60 nm ± 8 nm. NWs were mechanically detached from the substrate by means of sonication and dispersed in isopropyl alcohol (IPA). NWs were deposited onto a p++Si/SiO_2_ substrate by a technique of drop-casting. For suspended devices, the fabrication protocol includes a preliminary step, which involves the use of a sacrificial layer. For both device geometries, the sample was then patterned using standard e-beam techniques, using a positive polymethyl methacrylate (PMMA) e-resist. After the development and prior to metal evaporation (Ti/Au, 10/100 nm), the NW contact areas were passivated using a standard ammonium polysulfide (NH_4_)_2_Sx solution in order to promote the formation of low-resistance ohmic contacts. The fabrication protocols of the two device geometries are schematically depicted in Figure 1a (supported NW device) and in Figure 1b (suspended NW device). More detailed information about the fabrication protocol of suspended devices can be found in [57].

**Figure 2:**
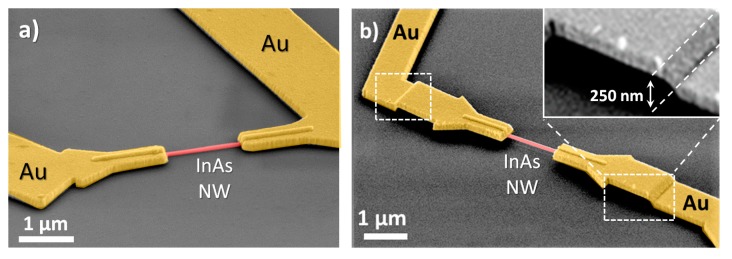
False color scanning electron micrograph of supported (**a**) and suspended (**b**) InAs nanowire-based devices, on a SiO_2_/p++Si substrate. In both images, the nanowires are red colored, while Ti/Au contact electrodes are yellow-colored. In device (**a**) the NW is in contact with the substrate, while in the device (**b**), it is suspended at about 250 nm over the substrate.

False color scanning electron micrographs of two of the fabricated devices are reported in Figure 2a (supported NW device) and 2b (suspended NW device), respectively. Preliminary characterization of the electrical transport properties of the nanowires used in this work was performed in vacuum at room temperature, allowing us to estimate the electron mobility *µ* ≈ 500 cm^2^/Vs and density *n*_e_ ≈ 10^18^ cm^−3^ (see Appendix B). After the fabrication of the nanowire-based sensors, prior to gas exposure, we tested the devices in the air at room temperature using a probe station. For gas sensing, the samples were mounted on a standard metal semiconductor package (TO5), and Au wire connections were realized using a wedge bonder.

We carried out sensing tests towards different gases, specifically humidity, ethanol, H_2_, and NO_2._ The tests were performed in a stainless-steel test chamber (volume of 1000 cm^3^) at a temperature of 20 °C, atmospheric pressure and using a constant gas flow of 300 cm^3^/min. We chose air as a carrier gas: the gas mixtures provided from certified bottles were diluted with the same carrier in order to move a step closer to the operating conditions of a real sensor. Gas mixtures of NO_2_ and H_2_ in synthetic air at ppm levels came from commercially available certified bottles. The humidity concentration was obtained using a bubbler filled with the de-ionized water and kept at room temperature (20 °C). The synthetic air carrier was fluxed through the bubbler to obtain humid air at almost 100% relative humidity at the ambient temperature [58]. Lower concentrations were obtained by volumetric mixing of humid air with synthetic air to obtain concentrations ranging from 5% to 70%. The same procedure was followed for the case of ethanol. In this case, the 100% concentration consisted of saturated vapor at 20 °C (the saturated vapor pressure at 20 °C was 46.63 Torr) and corresponded to approximately 30,000 ppm, while lower concentrations were obtained as explained before. Concentrations of ethanol, NO_2_, and H_2_ are reported in ppm; the % notation for relative humidity is used, as it is the most used in the literature on humidity sensors.

**Figure 3:**
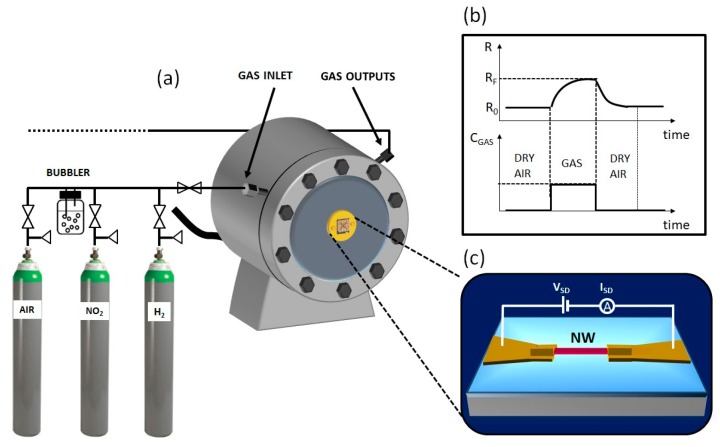
Schematic representation of the measurement setup used for sensing tests. (**a**) The setup consists of a 1000 cm^3^ stainless steel-test chamber, connected to a gas line fed from certified gas bottles, mixed through mass flow meters. A bubbler is connected to the line for obtaining humidity and ethanol air mixtures; (**b**) sketch of the transduced signal, i.e., the nanowire (NW) resistance variations as a function of time, related to the gas concentration inside the chamber during a test cycle; (**c**) scheme of the devices under test, showing the electric circuit for gas detection.

Figure 3 shows a scheme of the experimental setup used for gas sensing tests. The tests were carried out by applying a low and constant drain-source bias (in the range 0.01–0.04 V) and by continuously monitoring the electrical current across the nanowire, using a Keithley electrometer (Model 6514). The signal transduction relied on the modifications, which were induced in the current flow across the nanowire as a consequence of the adsorption of the gas molecules on its surface.

The sensing response was characterized by means of the following parameters, derived from resistance measurements: (i) Relative response, *RR*, which is calculated as *RR* = (*R*_F_ − *R*_0_)/*R*_0_, where *R*_F_ is the steady-state resistance when the nanowire is exposed to the gas mixture, and *R*_0_ is the steady state—or baseline—resistance when the nanowire is exposed to synthetic dry air only; (ii) Sensitivity, which is defined as the derivative of the calibration plot in the working point; (iii) Response (Recovery) time, which is defined as the time needed to reach 90% (70%) of the steady-state value in gas (air). Due to the physical constraints of the test chamber (gas flow 300 cm^3^/min and volume 1000 cm^3^), a complete exchange of the chamber atmosphere took more than three minutes, so shorter response and recovery times could not be evaluated in the present setup.

## 3. Results

The comparison between the performances of the supported and the suspended nanowire sensors was based on humidity tests, by evaluating their responses upon variations of the atmosphere from dry air to humid air in the range from 20% to 70% of relative humidity (r.h.). Each cycle included a period of 1 h, during which the sensors were exposed to a specific and constant humidity concentration, followed by a recovery period of 1 h, during which the sample was allowed to recover to the baseline resistance in dry air. The bias, applied to the source and drain contacts, was intentionally kept low, in order to avoid possible nanowire damage by self-heating. The baseline resistance of supported nanowires was typically in the range between 100 kΩ and 150 kΩ (measured with applied source-drain voltage *V*_DS_ = 0.04 V), and the baseline resistance of suspended nanowires was typically in the range from 30 kΩ to 50 kΩ (*V*_DS_ = 0.01 V).

**Figure 4:**
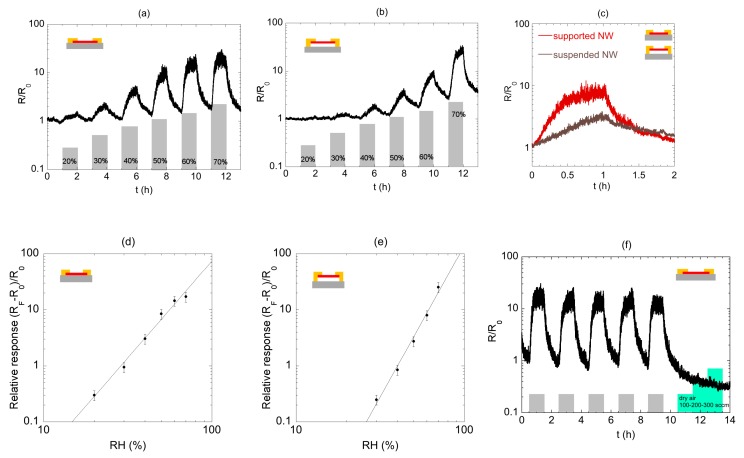
Dynamic variation of the normalized nanowire resistance towards changes in relative humidity (r.h.) from 20% to 70% for (**a**) supported nanowire and (**b**) suspended nanowire. The cycle time (humid air/dry air) is 2 h. (**c**) Normalized resistance for the two devices, for 50% r.h. (**d**) Calibration curve for supported device; extracted fit parameters (power law) are A = 9.06 × 10^‒6^ and B = 3.45. (**e**) Calibration curve for the suspended device; parameters of the power law in the fit are A = 3.18 × 10^‒5^; B = 3.15. (**f**) Repeatability of the resistance change when the substrate-bound NW device experience a change between dry air and 50% r.h. (gray area). Light-blue steps refer to change in the air flowing through the test chamber from 100 to 300 sccm.

The results are summarized in Figure 4. Both the device architectures showed a very good response towards humidity exposure (Figure 4a,b, reporting the normalized resistance versus time). In particular, during each sensing cycle, an increase in the device resistance was observed upon exposure to humidity, followed by a drop of the resistance to the baseline value during the recovery period (dry air flushing). Moreover, the maximum value of the resistance during each sensing cycle increased by increasing the value of the relative humidity (r.h.). The results showed that the supported NW (Figure 4a) was sensitive to humidity, even in the low concentration range. This was not the case for the suspended NW (Figure 4b): in this case, humidity detection became evident from 40% r.h. The comparison of the normalized resistances for the two sensors for 50% r.h. is shown in Figure 4c. The graph shows a higher response for supported NW, together with faster dynamics. It is noticeable that in the case of suspended NW, the steady state in 50% r.h. condition was not reached within 1 h. This was confirmed by the calculated response times (31 and 45 min for supported and suspended devices, respectively) and recovery times (13 and 24 min, respectively). To calculate the relative response for suspended devices, we made the assumption that the sensor resistance recovered to the initial value: we calculated the relative response using the baseline resistance in dry air at the beginning of the measurement, together with the resistance value at the end of the humid pulse of gas.

Figure 4d,e report the calibration plots of the relative response, *RR*, as a function of r.h. for the supported and the suspended devices, respectively. The data were fitted using a power law R = A × [C]^B^, where A and B are coefficients for the fit, and C is the humidity concentration in %. The limit of detection for the humidity, estimated considering three times the baseline noise (see ahead in the manuscript and Appendix C), was lower for the supported device (≈ 30%) than for suspended device (≈ 40%). The derivative of the calibration curve at 50% r.h, i.e., the Sensitivity S_50%_, was higher for supported NW, which also showed a higher relative response. At 70%, S_70%_ was higher for suspended NW, yet the relative response in this region was very similar for the two devices. For r.h. lower or equal to 60%, the relative response of substrate-bound NW was higher with respect to suspended one. These results suggested that suspended NW-based device did not allow an improvement of the sensing performances in the low concentration range (below 70%) and showed similar performances in the high concentration range. As a consequence, further investigations were focused on the supported NW device only. In order to validate the device as a gas sensor, we performed repeatability and flow tests. Figure 4f shows the behavior of the device resistance during repeated sensing cycles, using for each cycle the same relative humidity concentration (r.h. = 50 % in this specific plot). These short-term repeatability measurements showed no drop in sensor response towards humidity after five cycles, while long-term measurements go beyond the scope of the present paper. The same graph reports, starting from time t = 10 h, the resistance as a function of the gas flow used during the measurements. The resistance did not show any variation upon changes in the gas flow, which demonstrated that the recorded signal variations were due to the interaction of the NW to its environment rather than to case-dependent operational conditions.

**Figure 5:**
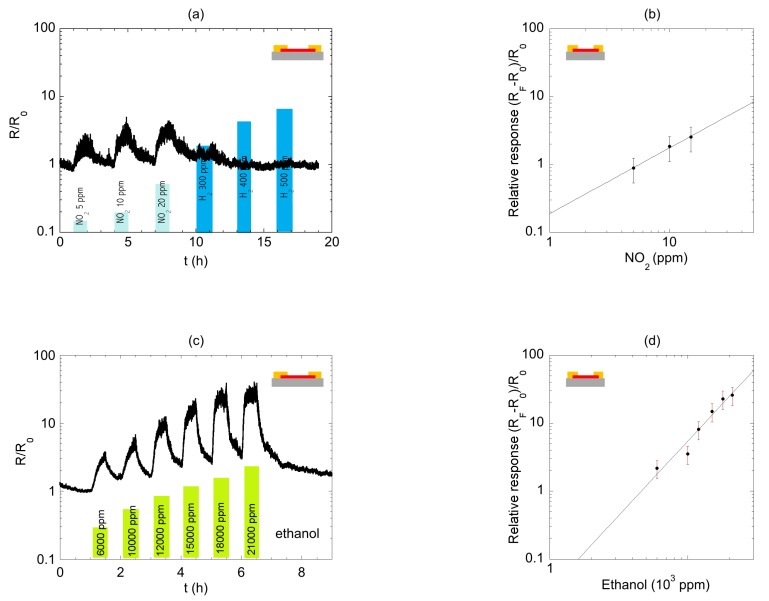
(**a**) Dynamic variation of the normalized resistance for supported nanowire (NW) towards NO_2_ (5–10–20 ppm in dry air) and H_2_ (300–400–500 ppm in dry air). (**b**) Calibration plot for NO_2_ detection; parameters of the power law in the fit are A = 0.19; B = 0.96. (**c**) Dynamic variation of the normalized resistance for supported NW towards ethanol (in the range 20% to 70%, corresponding to the range 6000 ppm–21000 ppm). (**d**) Calibration plot for ethanol detection; parameters of the power law in the fit are A = 0.002; B = 2.16.

The tests with the other target gases were focused on the substrate-bound device only. Figure 5a shows sensing tests with NO_2_ (5–10–20 ppm) and H_2_ (300–400–500 ppm). The device was quite insensitive to H_2_ and could detect concentrations down to 5 ppm of NO_2_. The calibration plot (Figure 5b) showed that the data were fitted by a power law. The extrapolation of the fit to the axis limit gave an indication of the lowest detectable NO_2_ concentration, which was equal to 1 ppm. Figure 5c reports the dynamic resistance variation associated with ethanol concentration varying from 20% to 70%. The calibration plot for ethanol (Figure 5d) showed that this gas could easily be detected in the selected concentration range.

By extrapolating the calibration fitting curves reported in Figure 4d and in Figure 5b,d to values lower than that measured, we could evaluate the limit of detection (LOD) of the substrate-bound nanowire device. To this aim, we assumed that the smallest discernible signal (x_gas_) emerges from—is not a random fluctuation of—the baseline (x_B_) when (x_ga_s-x_B_) is at least three times the noise of the baseline. This, following the simple procedure reported in detail in the Appendix C, allowed us to estimate the different LOD for the three analytes tested, namely, 30% for R.H., 4000 ppm for Ethanol, and 5 ppm for NO_2_.

Before the discussion of the observed results (see Section 4), here it is worth to analyze briefly the sources of electrical noise in our measurements due to equipment and thermal effect. First, the used electrometer accounted for a <2.6 mV rms noise in the voltage source, with an accuracy of 0.15 mV + V_0_ with the offset V_0_ assuming a maximum value of 10 mV depending on the used sensitivity, while the accuracy of the provided ohmmeter was 0.125 + 10 Ω (offset). Second, measurements were performed at room temperature: assuming that the active channel (nanowire) was coupled to two ideal electronic reservoirs having an occupancy following the Fermi-Dirac distribution, we roughly estimated the smearing of the distribution of ~24 meV (KT at 300 K). Thus, statistically, a flow of electrons in a broadened energy range occurred, raising thermal noise in the measurement. Third, thermal effects likely had an impact also on the frequency of adsorption/desorption of molecules on the surface of the nanowire. However, we also noticed that slightly different noise level –different signal-to-noise (S/N) ratio—was observed for different analytes. For instance, the signal for NO_2_ was more noisy respect to the one for ethanol. In order to rationalize this experimental evidence, we invoked two qualitative arguments, as discussed in the following. Preliminary, we noticed that the operation of our conductometric sensor was based on the physisorption of analyte molecules on the surface of the nanowire: molecules detaching from the surface introduced noise in the electrical signal. As the first argument, we pointed out that the gas flow inside the experimental chamber—particularly in the proximity of the nanowire—might display some turbulence, rather than being fully laminar. In general, the onset of turbulence in the gas flow around the sensor was driven by the small size of the sensor: in our case, the active device building block was the InAs nanowire with typical dimensions of approximately a few µm length and 60 nm diameter. The occurrence of turbulence might locally modify the concentration of gas molecules near the nanowire, and this generated some noise. Overall, this process yielded a higher level of noise when a few ppm of analyte were involved, respect to when thousands of ppm were considered. As a matter of fact, while the concentration of ethanol (low noise) ranged in thousands of ppm, the one of NO_2_ (high noise) was almost two orders of magnitude lower (5–15 ppm). As the second argument, we considered that, in first approximation, the noise associated to analyte molecules detaching from the nanowire surface—due to turbulence or thermal excitation—could be assumed as shot noise or Poisson noise, i.e., a type of noise which can be modeled by a Poisson process. This yielded to a signal-to-noise (S/N) ratio given by the square root of the “average number of events”. The latter in our case was proportional to the analyte concentration, thus the higher the concentration, the higher the S/N ratio: again, as a matter of fact, we experimentally observed higher S/N ratio for the analyte detected in the higher concentration (see Figure 5a,c).

## 4. Discussion

These results demonstrated single InAs NWs as reliable nanomaterials for the sensing of humidity, NO_2_, and ethanol molecules carried by synthetic air and adsorbed on the NW surface. This is likely due to the combination of two factors: the role of surface states and the electron surface accumulation layer [43,44], which likely makes InAs NWs very sensitive to the environment, and their relatively high surface to volume ratio. However, when it comes to the detection of ethanol and NO_2_, humidity variations may severely impact the results: for real sensor applications, that typically involve not-vanishing humidity, the latter should be kept under control.

The comparison between the performances of suspended and supported nanowire-based devices revealed that the availability of a slightly larger nanowire surface for gas interaction in suspended nanowire (six free facets instead of five in the surface-bound case), as well as the ideal geometry for gas flowing around the nanowires, are not reflected in higher sensor response, while they are linked to an increase of response and recovery times. Thus, there is no clear advantage in going through the complex sequence of fabrication steps needed to obtain suspended nanowire devices: the simpler supported geometry appears to be overall more effective.

A conclusive explanation for this experimental outcome likely deserves additional investigation efforts that go beyond the purpose of the present work. However, we tentatively speculate a non-trivial role of the O_2_ plasma processing step, which is necessary to remove the sacrificial layer during the fabrication of the suspended nanowire devices. In fact, while previous results of our group allow to rule out any direct impact of the O_2_ plasma treatment on the electrical transport properties of the nanowire [57], in the present experiment, we are not allowed to absolutely exclude that the oxygen plasma could impact detrimentally on the interaction between the nanowire surface and the sensed H_2_O molecules, making the suspended nanowire device less sensitive to relative humidity variations. As a matter of fact, several studies report evidence for O_2_ plasma-induced modification of an oxide surface [59,60]. Let us consider the seminal case of the silicon oxide surface. The SiO_2_ surface has a hydrophilic nature due to the hydrogen bonding between silanol groups on the surface and water molecules, and O_2_ plasma is known to remove organic contaminants, thus further enhancing hydrophilicity. However, this effect is temporary, as the surface tends to react with other chemical groups, such as hydrocarbon contaminants [61]. This further reaction likely induces hydrophobicity in the surface, detrimental to the mechanisms of surface-gas (H_2_O molecules) interactions. This tentative explanation is consistent with the low performances of the suspended nanowire-based device for low concentration range (below 70%), compared to the supported device, while the effect is reduced in the high concentration range (above 70%). Besides, we cannot exclude the occurrence of undetectable residual contaminations of hydrophobic crosslinked PMMA in the region underneath the nanowire, due to an incomplete removal by O_2_ plasma treatment: these organic contaminants could reduce the mass transfer and convection around the sensing element and, overall, enhance the hydrophobicity of the region underneath the nanowire. However, the comparison between supported and suspended nanowire-based devices deserve a brief further comment. The conducting building block is the same for the two kinds of device geometries, as both the suspended and supported nanowire-based devices were fabricated using NWs isolated from the same growth batch. Nevertheless, the fabrication protocols developed for the two device geometries are necessarily different, because suspended NW devices require additional fabrication steps with respect to the supported ones. In particular, a key ingredient for the realization of suspended devices is oxygen plasma treatment: while the specific conditions of this fabrication step have been tuned to minimize their impact on the transport properties of the nanowires, still, as a matter of fact, one should regard the suspended-nanowire-based sensor not exclusively as the suspended InAs nanostructure but as the entire device. The sensing tests towards humidity, NO_2_, and ethanol indicated that InAs nanowires were well suited to the detection of both reducing and oxidizing species in ambient air, and, despite the small dimensions of the active region, relevant signal variations related to the adsorption process could be easily detected.

Here, for completeness, we provide a comparison between the sensing performances observed in our InAs NW-based devices and the ones reported in the literature for semiconductor oxides. Since the literature on semiconductor oxides gas sensor is extremely wide, it is worth comparing the results of our work to those reported for individual nanowires of semiconductor metal oxides (MOX). MOX single nanowire-based devices are typically exploited for sensing applications at temperatures well above room temperature [62] or, alternatively, self-heating effects are used to heat locally the nanostructure [63]. The use of four contact measurements is usually reported for MOX NW-based devices (see, for instance, Reference [64]). As a matter of fact, the realization of affordable electrical contact on MOX nanowires is extremely challenging, as one has to face the need to exclude very high contact resistances that can be severely detrimental to the electrical response of the device. The technology of ohmic electrical contacts on III-V semiconductor materials, e.g., InAs is instead fully developed both in bulk and at the nanoscale, in particular, for the case of individual nano-objects, such as nanowires [65], and at the same time, InAs NW sensors are fully compatible with room temperature operation. Stated this, let us consider a few specific cases of study. Regarding ethanol and humidity detection, we achieved impressive results using MOX NW sensors: however, the sensors were operated well above not room temperature. In fact, for ethanol detection, (e.g.,) SnO_2_ nanowires were kept at 300 °C [66], while for humidity detection, SnO_2_ nanowires were operated in a self-heating pulsed mode [67]. In the following, we focused on room temperature operation of MOX, in order to deal with conditions comparable to those used in this work. Individual SnO_2_ nanowires showed a relative response of 6 to 50% r.h. [68], that is lower with respect to the relative response displayed by our InAs NW-based sensors. Concerning NO_2_ detection (see for instance Reference [34]), individual SnO_2_ nanowires displayed lower response (0.2 to 4 ppm) with respect to InAs NW-based sensors (0.9 to 5 ppm), while individual ZnO nanowire showed much higher response (i.e., exceeding two orders of magnitude change): however, it has to be said that the ZnO sensor has very poor recovery, as it works in dark. These and many other works indicate that InAs nanowires, used as building blocks for gas sensing applications, may enable sensing performances that are comparable or even overcome the ones reported for MOX NWs. Of course, the performances of the two families of nanomaterials can be extremely different according to different parameters, such as the analyte and the operating conditions (temperature), to name a few.

The effect observed for all the detected species is a resistance increase, indicating that the InAs nanowire-based sensors developed in this work likely display poor selectivity, as typically occur for the case of their metal oxide counterparts [68]. These results are partly in apparent contrast to some other findings reported in the literature [46,48]. For oxidizing gases, we expect that NO_2_ is adsorbed as NO_2_^-^, trapping an electron on the nanowire surface, thus electrically affecting the conduction of electron in the nanowire channel. This hypothesis is in agreement with the observed reduced current flowing into the InAs device, and it confirms other observations, in an inert atmosphere, reported in the literature [49]. The sensor does not show a sizeable response to H_2._

Regarding the effect of water absorption on InAs nanowires, the mechanisms of interaction are instead still debated. Du et al. [48] carried out experiments of water absorption on InAs NW field effect transistors, using inert gas as a carrier (He). Their investigation of the gate voltage response suggests that water absorption induces both a reduction in the carrier concentration and an enhancement in the carrier mobility and that the observed global behavior, i.e., a current increase, is a balance between the two effects. Actually, similar phenomenology was reported for ethanol. On the contrary, Zhang et al. [48], while performing experiments similar to those of Du et al., claimed that the effect of H_2_O adsorption on the InAs NW transport properties is the increase of the electron density in the channel, without a major role of mobility. Even though in both cases, the net effect of water absorption on InAs NWs is an increase in the measured current, the results suggest the presence of competing mechanisms.

In our case, it is worth noting that the direct comparison with the results presented by Du and Zhang can be misleading and needs some additional reasoning. On the one hand, our results are consistent with the hypothesis of a reduced carrier density in InAs NWs in the presence of air and other adsorbates, observed by Du et al. The resistance of our nanowires in dry air is in fact quite higher with respect to the behavior in vacuum (see Appendix B). Ullah et al. [47] also demonstrated that the adsorption of polar species, such as H_2_O and alcohols, leads to a decrease in the carrier density in the InAs channel and a decrease in the drain-source current at zero bias. The net effect observed by these authors upon exposure to N_2_ or ethanol was a reduction of the drain-source current: this is consistent with our experimental outcomes.

The mentioned studies [46,47] were performed in an inert atmosphere or in a vacuum: as claimed in the same studies, inert gases do not interact at all with the surface of InAs NWs, and their effect on the sensor behavior is equivalent to the vacuum conditions. Instead, the same does not occur if the sensor is operated in air, a more appropriate environment for testing the sensor in operating conditions is closer to real life. In our case, the baseline signal is acquired in dry air, and first interaction with the nanowire surface is already established during the stabilization phase, which contributes to the global balancing of the effects throughout the experiment. The target gases are then injected when the surface is already in equilibrium with the dry air atmosphere, a condition that differs substantially from the one adopted in previous studies. The increase in the concentration of polar species, such as H_2_O, in a dry air background likely further reduces the carrier density in the nanowire. Decrease of carrier density is likely the reason for the current decrease observed both for reducing and oxidizing gases. However, this thesis would require further experiments to be verified.

## 5. Conclusions

In conclusion, we have reported the use of individual InAs nanowires for the realization of conductometric sensors for the detection of different chemical species diluted in synthetic air. We investigated the performances of two different types of device architectures towards humidity: supported geometry, where the nanowire is in direct contact with the substrate, and suspended geometry, where the nanowire is suspended at approximately 250 nm over the substrate. The results show that the availability of a larger area of the nanowire surface for gas interaction in suspended nanowires is not reflected in higher sensor response. The performance of the supported nanowire-based devices was then tested upon different target gases, showing a clear response to NO_2_ and Ethanol diluted in dry air. Differently from most of the previous experiments that were performed with inert atmosphere as a gas carrier, in our case, the effect observed for all the detected species was a resistance increase: the current results provide new insights in the understanding of the gas sensing mechanisms in InAs nanowires in real operating conditions. However, here it is worth clarifying that the study of the effect of humidity goes beyond the scope of the present paper, and the absence of humidity as a vector for different analytes during our experiments sets the distance between our experiments and the real-life conditions. At a time, this work sets the basis for the full study of the impact of humidity as a simulator of real-life conditions for the detection of gases by InAs nanowire-based devices.

## Figures and Tables

**Figure A1:**
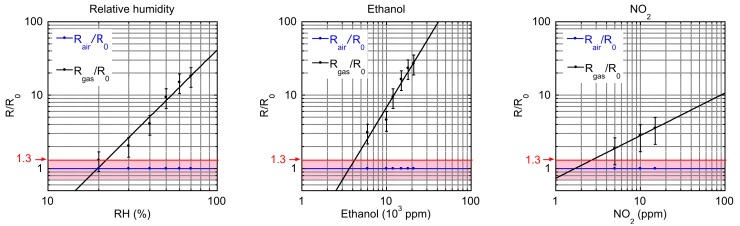
A graphical estimate of the limit of detection (LOD) of different analytes in single InAs nanowire-based sensor: the LOD corresponds to the abscissa at the intersection between the horizontal line at 1.3 and the Rgas/R0 fitting curve for each analyte.

## Appendix A

We have fabricated 32 supported NW-based devices, and we have preliminarily tested all of them in a vacuum (approx. 10^−2^ mbar) at 300 K: we found that all were electrically conducting and display resistance in the range from 30 kΩ to 80 kΩ. This preliminary characterization was done using a set up optimized for measuring individual nano-objects but not suitable for gas sensing experiments. The latter initially did not match the level of electrical isolation and the absence of a ground loop that is typically required for safe measurements of our nanowires. The optimization process was not-trivial and had a dramatic impact on our portfolio of devices: out of 32 fabricated devices tested in a vacuum, unfortunately, only three survived to the preliminary test needed for the optimization of the test chamber. Using these devices, we realized the main experiments whose results are reported in the manuscript. Regarding the suspended NW-based devices, where the bottleneck was the development of an affordable device fabrication protocol: this involves additional steps with respect to the protocol for supported devices. In particular, we had to optimize the oxygen plasma conditions in order to remove the resist without affecting the transport properties of our NWs. Here, the scenario is similar to the one described for supported NW-based devices. Out of approximately 15 iterations of device fabrication steps, and of further minor issues dealing with the sensing chamber, we tested in a vacuum and used just two devices for our experiments. Concerning the variance between samples, we observed a 20% variation between different samples, calculated on the relative response to 50% relative humidity. The error bars reported in the calibration curves were calculated using the results of repeatability tests shown in Figure 4f and based on the noise level observed upon steady-state gas flow.

## Appendix B

Preliminary to the experiments reported in this work, in order to characterize the pristine electrical transport properties of the InAs nanowires used in this work, we realized a set of field effect devices using nanowires belonging to the same growth batch of the nanowires used for the sensing experiments. The fabrication process was very similar to those used for the fabrication of supported sensor reported in Section 2. Two metal electrodes were used as source and drain, while the SiO_2_ /Si-p++ substrate was used as a gate electrode in a standard back-gate configuration. The electrical properties were tested at room temperature and in a vacuum (pressure of the order of 10^-2^ mbar). The DC source-drain voltage (*V*_DS_) was supplied by the DC output of an SR380 Lock-in amplifier, while a DC voltage ranging from –30 V to 30 V was applied to the back gate (*V*_G_), using a Yokogawa 7651 Programmable DC Source. The current signal across the NW channel was then detected using a current preamplifier. The room temperature electrical transport characteristics of the investigated devices indicated nanowire resistance in the range from 30 kΩ to 80 kΩ. Transconductance measurements revealed that our InAs semiconductor nanowires were n-type; besides, we extracted the electron mobility and density using a standard charge-control method accounting for the silicon oxide thickness and for the length and width of the nanowire conducting channel between source and the drain electrode. The average calculated electron mobility was about 500 cm^2^/(V·s), and the electron density was of the order of 10^18^ cm^‒3^. We noticed that the systematic investigation of the sensing performance of our devices operated as field effect transistors goes beyond the purpose of the present work, while may represent a challenging task for future investigations.

## Appendix C

Following the literature [69,70], we adopted a LOD defined as follows. We assumed that the smallest discernible signal (x_gas_) emerged from (is not a random fluctuation of) the baseline (x_B_) when (x_gas_-x_B_) is at least three times the noise of the baseline. Now, in the plot below, we showed the baseline resistance (in the air) normalized to one, R_air_/R_0,_ and we reported the sensor resistance in gas, R_gas_/R_0_. Actually, the noise of the sensor baseline (R_air_/R_0_) was ≈ 0.1 during all tests: by taking three times this value, we got 0.3. We summed this value to the normalized baseline resistance, and we got the horizontal line at 1.3 depicted in pink in the graphs in Figure A1: this line marks the lower threshold for a discernible signal. The intercept between this line and data extrapolation fit for each specific analyte returned the LOD for that analyte. In particular, we got 30% for r.h., 4000 ppm for Ethanol, and 3 ppm for NO_2_. In the latter case, to take into account the high noise and low S/N ratio, we could artificially raise the LOD for NO_2_ to 5 ppm.
